# Nanosized Mesoporous Bioactive Glass/Poly(lactic-co-glycolic Acid) Composite-Coated CaSiO_**3**_ Scaffolds with Multifunctional Properties for Bone Tissue Engineering

**DOI:** 10.1155/2014/323046

**Published:** 2014-03-02

**Authors:** Mengchao Shi, Dong Zhai, Lang Zhao, Chengtie Wu, Jiang Chang

**Affiliations:** State Key Laboratory of High Performance Ceramics and Superfine Microstructure, Shanghai Institute of Ceramics, Chinese Academy of Sciences, Shanghai 200050, China

## Abstract

It is of great importance to prepare multifunctional scaffolds combining good mechanical strength, bioactivity, and drug delivery ability for bone tissue engineering. In this study, nanosized mesoporous bioglass/poly(lactic-co-glycolic acid) composite-coated calcium silicate scaffolds, named NMBG-PLGA/CS, were successfully prepared. The morphology and structure of the prepared scaffolds were characterized by scanning electron microscopy and X-ray diffraction. The effects of NMBG on the apatite mineralization activity and mechanical strength of the scaffolds and the attachment, proliferation, and alkaline phosphatase activity of MC3T3 cells as well as drug ibuprofen delivery properties were systematically studied. Compared to pure CS scaffolds and PLGA/CS scaffolds, the prepared NMBG-PLGA/CS scaffolds had greatly improved apatite mineralization activity in simulated body fluids, much higher mechanical property, and supported the attachment of MC3T3 cells and enhanced the cell proliferation and ALP activity. Furthermore, the prepared NMBG-PLGA/CS scaffolds could be used for delivering ibuprofen with a sustained release profile. Our study suggests that the prepared NMBG-PLGA/CS scaffolds have improved physicochemical, biological, and drug-delivery property as compared to conventional CS scaffolds, indicating that the multifunctional property of the prepared scaffolds for the potential application of bone tissue engineering.

## 1. Introduction

As a promising material for bone tissue engineering, CaSiO_3_ (CS) has been widely studied for years due to its distinct bioactivity and degradability [1–3]. Up to now, CS powders, coatings, and scaffolds have been prepared by various methods and the *in vitro* and *in vivo* biological properties have been studied [2, 4–6]. Among them, CS scaffolds with a porous structure which provides sufficient space for cell migration and ingrowths have gained much attention for bone regeneration application. However, the inherent brittleness and the high local pH environment that resulted from the high ionic dissolution rate of the prepared CS scaffolds limited their further application as bone tissue engineering scaffolds [7]. In order to solve these problems, polymers such as PLA (polylactic acid) and PCL (polycaprolactone) have been used to improve the mechanical property and to decrease degradation rate of CS scaffolds [8–10]. Previous studies demonstrated that with PLGA (poly(lactic-co-glycolic acid)) coating on the surface of pore walls and the CS scaffolds possess better compressive strength and the pH value of biological solution reduced during the degradation of scaffolds [11]. However, as the pore walls of porous CS scaffolds were covered by PLGA coatings, the bioactivity of CS scaffolds was inhibited due to the shielding effect of PLGA on the bioactive inorganic phase.

Ideal scaffolds for bone tissue engineering should have multifunctional properties, such as proper mechanical strength, good bioactivity, proper degradation rate, and even drug-delivery ability [12, 13]. More and more studies have shown that drug and growth factor delivery via porous scaffolds may play an important role to deal with the potential infections and to accelerate the osteogenesis and angiogenesis process during bone regeneration [14–16]. However, how to prepare multifunctional scaffolds with these specific properties still remains a significant challenge.

In the past several years, a new class of biomaterials, mesoporous bioactive glasses (MBG), has attracted much attention for bone regeneration and drug delivery [17]. They have highly ordered mesoporous channel structure, large surface area, and variable pore volume. These features greatly enhanced their apatite-mineralization ability as well as drug-delivery ability [18, 19]. Due to the advantages of MBG, we have previously prepared pure MBG scaffolds which combine hierarchical large pore (300–500 *μ*m) and well-ordered mesopores (5 nm) for bone tissue engineering and drug delivery [20]. However, the shortcoming of the prepared MBG scaffolds is their low mechanical strength since they cannot be sintered at high temperature (no more than 700°C) [21] and the mesopore structure will be damaged if the sintering temperature is higher than 700°C. As we mentioned above, CS scaffolds possess distinct bioactivity and biopolymer-modified CS scaffolds that have excellent mechanical strength [11]. For these reasons, it is assumed that the incorporation of MBG particles into biopolymers for coating CS scaffolds may combine the bioactivity and drug-delivery property of MBG with the improved mechanical strength of biopolymer modification. Therefore, the aim of this study is to prepare CS scaffolds with a coating layer of nanosized MBG (NMBG) particles/PLGA composite and to further investigate the effect of NMBG/PLGA coating on the mechanical property and apatite-mineralization activity of the scaffolds and the proliferation and differentiation of bone-forming cells as well as the drug-delivery property of CS scaffolds.

## 2. Materials and Methods

### 2.1. Preparation and Characterization of Nanosized Mesoporous Bioactive Glass Particles

Nanosized mesoporous bioactive glass (NMBG) powders were synthesized using cetyltrimethylammonium bromide (CTAB) as template, in which 6.6 g CTAB was firstly dissolved in 600 mL of distilled water with 12 mL of ammonia water. After stirring for 1 h at 37°C, 30 mL tetraethyl orthosilicate (TEOS) and 31.21 g Ca(NO_3_)_2_·4H_2_O were added to the solution and stirred for 6 h. The products were collected by vacuum filtration and washed by distilled water and ethanol for 3 times. Ethanol mixed with 1% HCl solution was used to wash the powders to remove CTAB. After dried at 60°C for 24 h, the powders were calcined at 550°C for 2 h. The NMBG powders were characterized by X-ray diffraction (XRD) (D/Max 2550V, Rigaku Japan), scanning electron microscopy (SEM) (JSM-6700, JEOL, Japan), and transmission electron microscopy (TEM) (2100F, JEOL, Japan). The specific surface area, mesopore size distribution, and pore volume were determined by N_2_ adsorption-desorption isotherms (Micromeritics Tristar 3000).

### 2.2. Preparation and Characterization of Calcium Silicate (CS) Scaffolds, PLGA/CS Scaffolds, and NMBG-PLGA/CS Scaffolds

CS scaffolds were prepared by polyurethane template method according to our previous study [22], CS powders (<40 um) were added to polyvinyl alcohol (PVA) aqueous solution (6% Wt) and stirred to get well-distributed slurry. Polyurethane sponges (PS) with a size of Ø6 × 6 mm and pore size of 300–500 *μ*m were prepared and immersed in a glass beaker containing the slurry and compressed with a glass stick to force the slurry to migrate into the pores of the foams. The struts of the foams were uniformly coated with ceramic slurry, while the pores were kept open. Then the sponges were transferred to a petri dish to dry at 60°C for 24 h. Once the samples were completely dry, they were calcined at 300°C for 2 h to remove the PS and then sintered at 1250°C for 3 h to obtain the CS scaffolds. The shape of scaffolds remained the same after sintering.

To prepared NMBG-PLGA/CS scaffolds, 2 g of PLGA (copolymer ratio of PLA : PGA 75 : 25) was dissolved in 20 mL acetone and stirred at room temperature. Different amounts of NMBG powders (0, 0.2, 0.6 or 1 g) were added to the solution under stirring. Then the CS scaffolds were immersed into the solution for 2 h while continuing to stir. The coated scaffolds were dried at room temperature overnight and then at 60°C for 24 h to remove the residual solvent. As a result, the PLGA/CS scaffolds (without NMBG particles), 10% NMBG-PLGA/CS, 30% NMBG-PLGA/CS, and 50% NMBG-PLGA/CS scaffolds were prepared. The morphology and pore structure of the prepared scaffolds were observed by SEM.

### 2.3. The Effect of NMBG Particles on the Mechanical Strength of Scaffolds

The compressive strength of scaffolds was tested using a universal mechanical machine at 0.5 mm/min crosshead speed (Shimadza AG-SKN, Japan). Six samples from each group were tested to obtain an average value.

### 2.4. The Effect of NMBG Particles on Apatite Mineralization of the Scaffolds in SBF

The assessment of *in vitro* bioactivity of the five types of scaffolds was carried out in simulated body fluids (SBF) solution. The SBF solution has a composition and ionic concentration similar to that of human plasma. Each scaffold was soaked in 10 mL of SBF solution in a polyethylene bottle at 37°C for 1, 3, 7, and 14 days. SBF solution was refreshed every other day. Then the scaffolds were collected, washed gently with distilled water, and dried at 60°C. The sizes of all scaffolds were around Ø6 × 6 mm as they were prepared. SEM and electron dispersive spectrometer (EDS) (INCA Energy, Oxford Instruments, UK) were used to examine the mineralized apatite on the surface of the scaffolds. The ion concentration (Ca, P, and Si) of the solution taken after soaking was tested by ICP-AES (Perkin-Elmer Optima 7000DV).

### 2.5. The Attachment, Proliferation, and Alkaline Phosphatase (ALP) Activity of MC3T3 Cells on Scaffolds

MC3T3 (MC3T3-E1 Subclone 14) cells were purchased from cell bank, Chinese Academy of Sciences. The 4th passage of MC3T3 was used for the evaluation of interaction of cells with the CS, PLGA/CS, and NMBG-PLGA/CS scaffolds including the attachment, proliferation, and alkaline phosphate (ALP) activity.

For evaluation of cell attachments, five kinds of scaffolds were sterilized in autoclave for 30 min and dried under 60°C. MC3T3 cells were cultured on scaffolds that were placed in 48-well culture plate at an initial density of 1 × 10^4^ cells/cm^2^. The cells were then incubated for 24 h in *α*-MEM culture medium supplemented with 10% FCS in humidified culture conditions. Then scaffolds were removed from the culture wells, rinsed with PBS, and fixed with 1.25% glutaraldehyde. The fixative was removed by washing with buffer containing 4% (w/v) sucrose in PBS and postfixed in 1% osmium tetroxide in PBS followed by sequential dehydration in graded ethanol. The specimens were dried in hexamethyldisilazane (HMDS) for 30 min before coating with gold for SEM analysis according to our previous publication [23].

For investigation of the proliferation of MC3T3 cells on the scaffolds, MTT assay was performed in triplicate according to our previous study protocol [24]. This assay is based on the cleavage of MTT into insoluble formazan crystals by the mitochondrial enzymes of the viable cells. Briefly, MC3T3 cells were seeded on these scaffolds and cultured in growth medium for 1, 3, and 7 days. 40 *μ*L of 0.5 mg/mL of MTT solution was added with 360 *μ*L growth medium at each time point. After incubated for 4 h, the medium was removed and the formazan product was dissolved in 200 *μ*L of dimethyl sulfoxide (DMSO). An aliquot of 100 *μ*L was taken from each well and transferred to a fresh 96-well plate. The absorbance was measured at *λ* = 590 nm on a microplate reader. All the results were demonstrated as the optical density values minus the absorbance of blank wells.

The effect of NMBG on the early osteogenic differentiation of MC3T3 cells on the scaffolds was performed to test their ALP activity assay by using PNPP method [25]. Cells were seeded at a concentration of 1 × 10^4^ cells/cm^2^ onto each scaffold placed individually in a 48-well plate. The cells were left to grow for 7 and 14 days at 37°C in a humidified atmosphere of 5% CO_2_. Aliquots of cell lysates were incubated with reaction solution (containing 2-amino-2-methyl-1-propanol, MgCl_2_, and p-nitrophenylphosphate) at 37°C for 30 min. The conversion of p-nitrophenylphosphate to p-nitrophenol was stopped by adding NaOH, and the absorbance at 405 nm was measured with a spectrophotometer (UV-Vis 8500, Shanghai). The ALP activity was normalized by total intracellular protein contents.

### 2.6. Loading and *In Vitro* Release of Ibuprofen (IBU) for NBMG-PLGA/CS Scaffolds

2 g of IBU was firstly dissolved in 50 mL hexane. 0.5 g NMBG powders were added to the solution and stirred for 24 h. Then the powders were centrifuged at 300 r/min, washed with distilled water, and dried at 60°C. The loading amount of IBU was determined by thermogravimetry (TG). The IBU-NMBG powders were added to the PLGA solution to prepare the 10%, 30%, and 50% NMBG-PLGA/CS scaffolds as described in Section 2.2. To test the release of IBU from scaffolds, each scaffold was soaked in 10 mL of PBS solution at 37°C on a shaking bed. The release medium was collected at the predetermined time intervals and replaced with fresh PBS solution. Then the released IBU from scaffolds was monitored by UV-Vis analysis.

### 2.7. Statistical Analysis

All the data were expressed as means ± standard deviation (SD) and were analyzed using one-way ANOVA with a post hoc test. A *P* value <0.05 was considered statistically significant.

## 3. Results

### 3.1. Characterization of NMBG Powders

**Figure 1:**
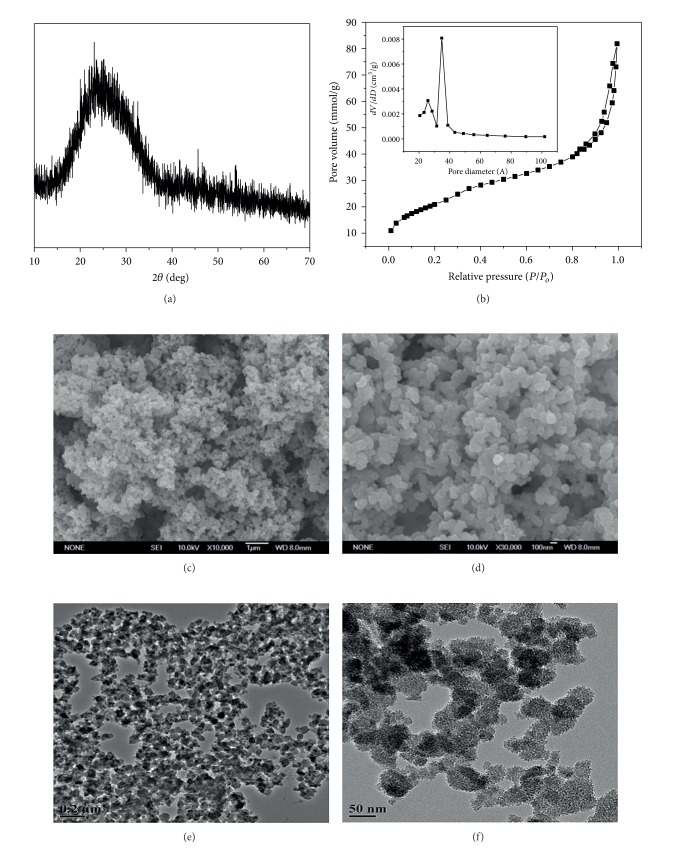
(a) XRD; (b) N_2_ adsorption-desorption isotherm; ((c), (d)) low and high magnification SEM images; and ((e), (f)) low and high magnification TEM images for NMBG powders.

The XRD pattern (Figure 1(a)) shows that there were no distinctly sharp characteristic peaks except for a wide peak at 2*θ* = 20–30°, suggesting that the prepared NMBG particles are amorphous. The SEM (Figures 1(c) and 1(d)) analysis shows that the size of the prepared NMBG powders is in the range of 50–100 nm. There are obvious nanopores in the inside of NMBG powders (Figures 1(e) and 1(f)). The N_2_ adsorption-desorption analysis presented a typical Type IV isotherm pattern (Figure 1(b)), which revealed that the NMBG powders possess mesoporous structure [26]. The mesopore distribution of the NMBG powders was around 3.5 nm. The specific surface area calculated by BET method was around 76.53 m^2^/g.

### 3.2. Characterization of CS Scaffolds, PLGA/CS Scaffolds and NMBG-PLGA/CS Scaffolds

**Figure 2:**
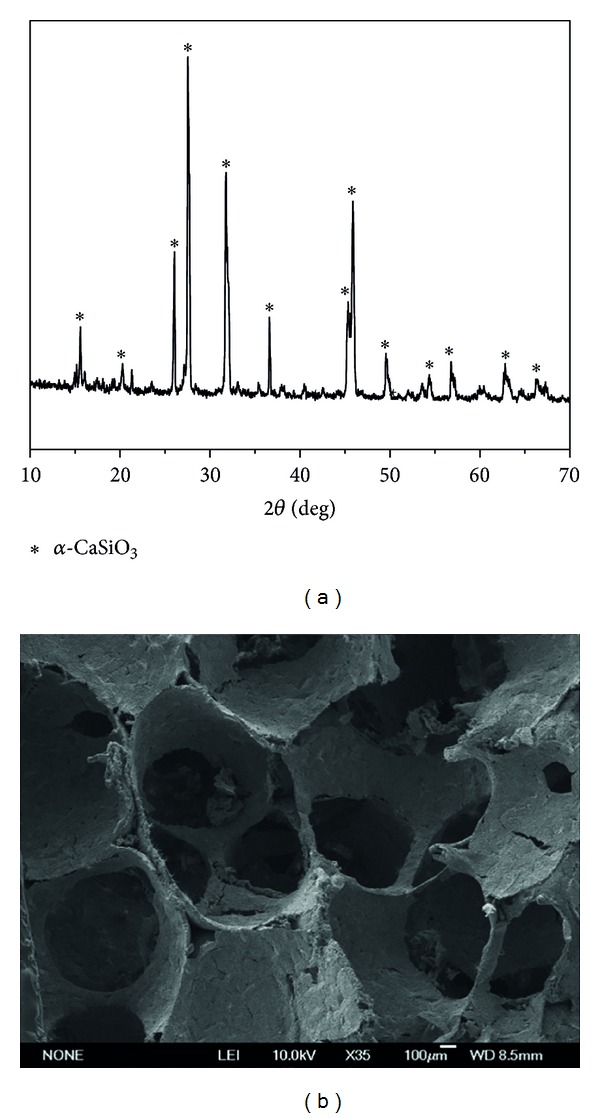
(a) XRD and (b) SEM analysis for CS scaffolds.

The prepared CS scaffolds present pure pseudowollastonite (*α*-CaSiO_3_) phase structure by XRD analysis (Figure 2(a)).  Figure 2(b) shows the representative SEM image of the CS scaffolds with interconnected macroporous networks and the pore size is in the range of 300–500 *μ*m.

**Figure 3:**
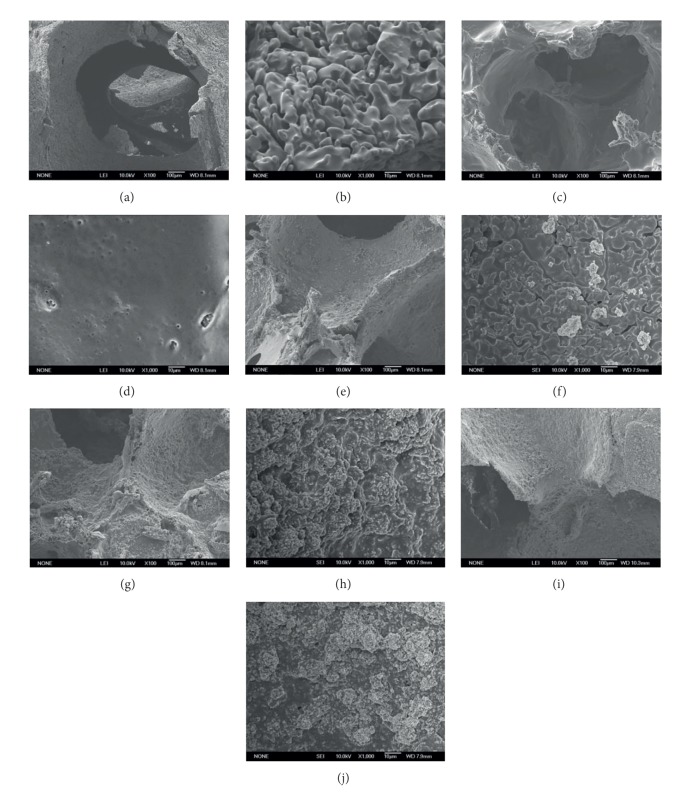
SEM images of CS ((a), (b)), PLGA/CS ((c), (d)), 10% NMBG-PLGA/CS ((e), (f)), 30% NMBG-PLGA/CS ((g), (h)), and 50% NMBG-PLGA/CS scaffolds ((i), (j)). ((a), (c), (e), (g), and (i)) low magnification; ((b), (d), (f), (h), and (j)) high magnification.

PLGA-coated CS scaffolds still maintain the nearly same structure (Figures 3(c) and 3(d)) while the surface becomes smoother. Figures 3(e), 3(g), and 3(i) presented the low magnification images of 10%, 30%, and 50% NMBG-PLGA/CS scaffolds, and Figures 3(f), 3(h), and 3(j) presented the high magnification images. As the contents of NMBG particles increased, the particles were mostly embedded in the pore walls of CS scaffolds, but the surface of pore walls became rougher. All these scaffolds remain the interconnected macroporous network (Figure 3).

### 3.3. The Effect of NMBG Particles on the Mechanical Strength and Apatite Mineralization of Scaffolds

**Figure 4:**
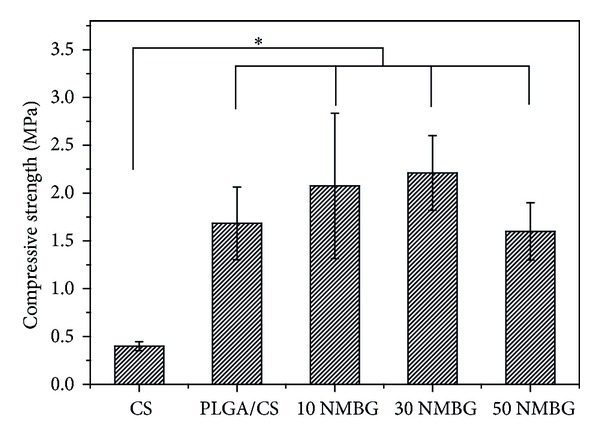
The compressive strength analysis for different scaffolds.

Figure 4 shows the compressive strength of the CS, PLGA/CS, and NMBG-PLGA/CS (10, 30, and 50% NMBG) scaffolds. It can be seen that PLGA coating significantly improves the mechanical property of CS scaffolds. The addition of NMBG powders has no distinct effect on the mechanical strength of scaffolds; however, three NMBG-PLGA/CS scaffolds with different contents of NMBG have significantly improved mechanical strength compared to pure CS scaffolds.

**Figure 5:**
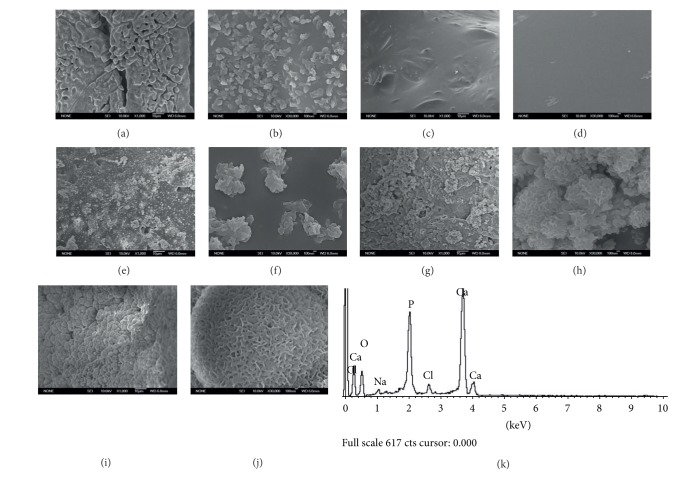
Apatite mineralization on the surface of scaffolds after soaking in SBF for 3 days. ((a), (b)) CS scaffolds, ((c), (d)) PLGA/CS scaffolds, ((e), (f)) 10% NMBG-PLGA/CS scaffolds, ((g), (h)) 30% NMBG-PLGA/CS scaffolds, ((i), (j)) 50% NMBG-PLGA/CS scaffolds, and (k) EDS analysis for 50% NMBG-PLGA/CS scaffolds.

Figure 5 presents the SEM images of five scaffolds after soaked in SBF for 3 days. For CS scaffolds, some apatite microparticles were deposited on the surface of pore walls (Figures 5(a) and 5(b)). For the PLGA/CS scaffolds (Figures 5(c) and 5(d)), the surface remained smooth and there were no deposited apatite particles. However, there were some apatite particles on the surface of 10% NMBG-PLGA/CS scaffolds (Figures 5(e) and 5(f)). A layer of apatite particles composed of worm-like microcrystals was found on the surface of both 30% NMBG-PLGA/CS and 50% NMBG-PLGA/CS scaffolds (Figures 5(g), 5(h), 5(i), and 5(j)). The EDS analysis exhibits that the ratio of Ca/P for the formed apatite is around 1.67.

**Figure 6:**
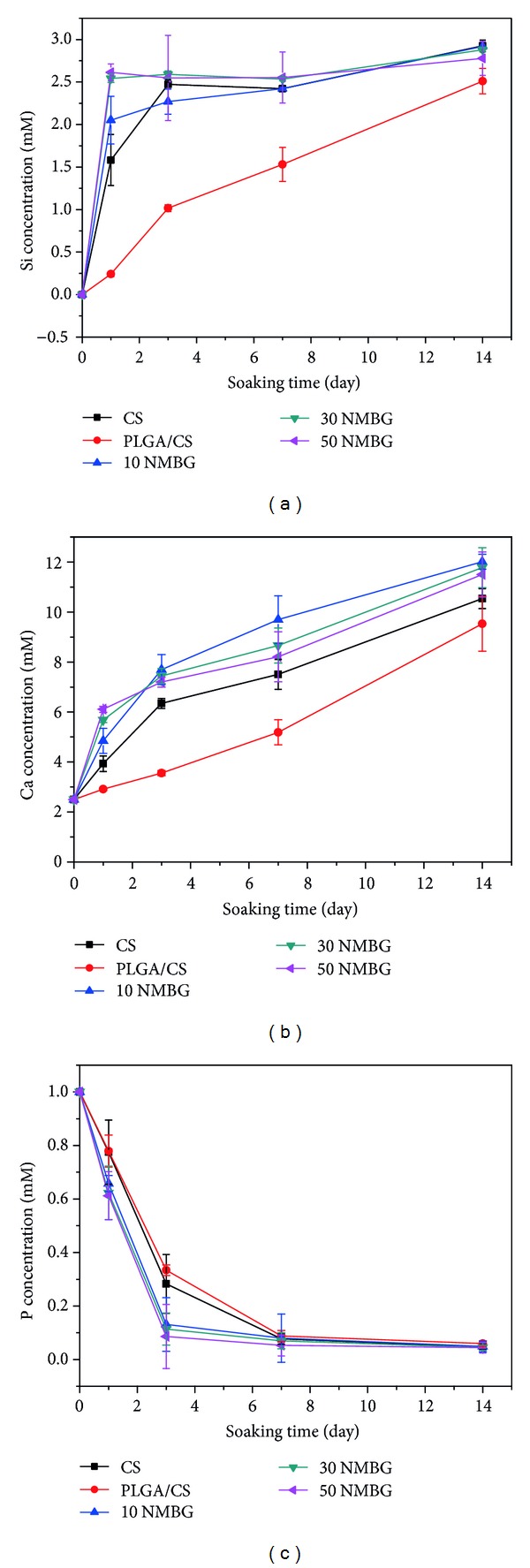
The change of (a) Si, (b) Ca, and (c) P ions in SBF solution after soaking the scaffolds for different times.

Figure 6 shows the change of Si, Ca, and P ions in SBF solution after soaked the scaffolds in SBF. The concentrations of Si and Ca in SBF for NMBG-PLGA/CS scaffolds are obviously higher than CS and PLGA/CS scaffolds (Figures 6(a) and 6(b)), but P concentrations are lower (Figure 6(c)).

### 3.4. The Attachment, Proliferation, and Alkaline Phosphatase (ALP) Activity of MC3T3 Cells on Scaffolds

**Figure 7:**
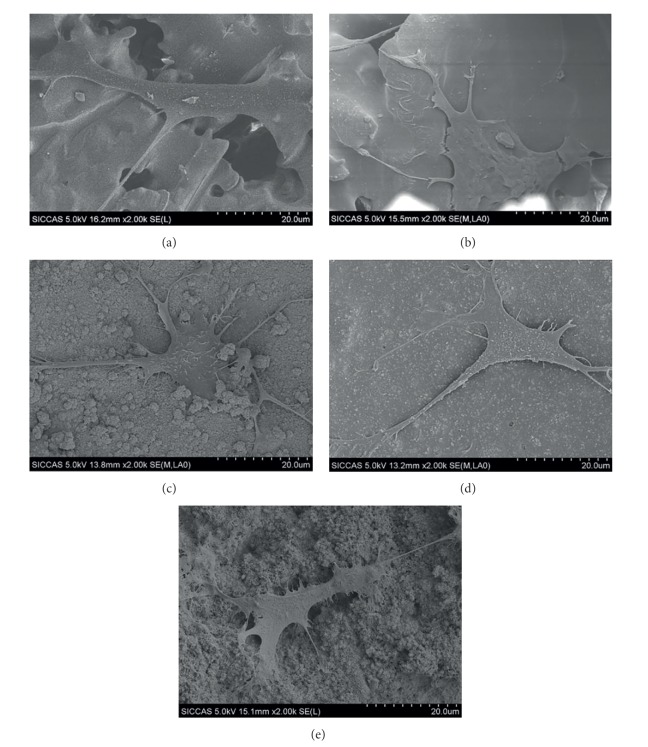
Cell attachments on (a) CS, (b) PLGA/CS, (c) 10% NMBG-PLGA/CS, (d) 30% NMBG-PLGA/CS, and (e) 50% NMBG-PLGA/CS scaffolds.

**Figure 8:**
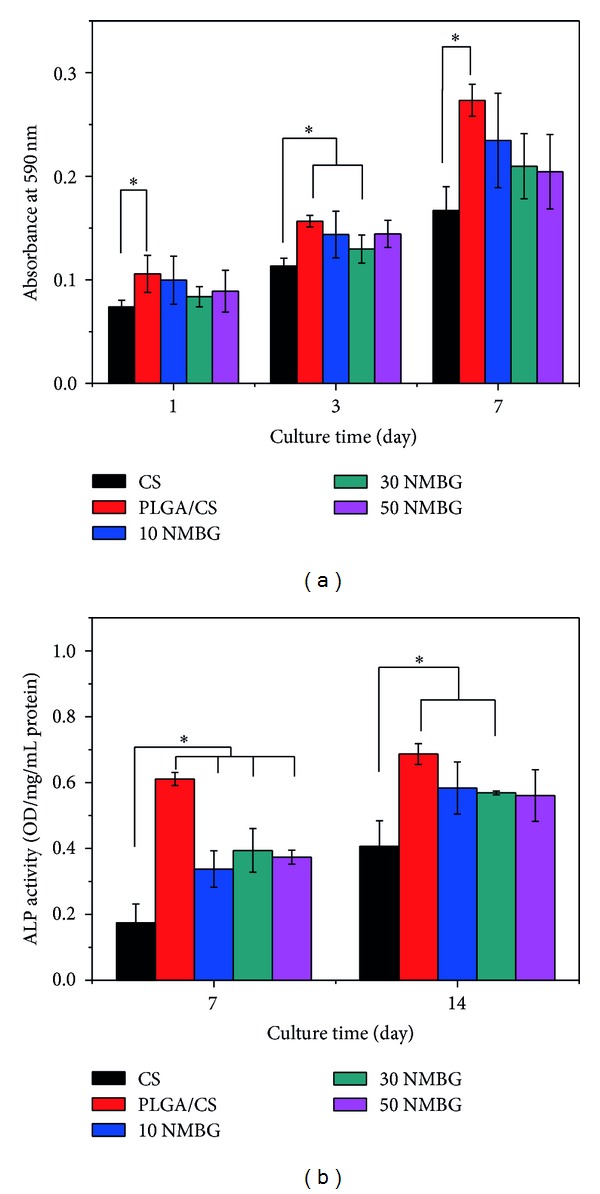
(a) The proliferation and (b) alkaline phosphatase (ALP) activity of MC3T3 cells on the five scaffolds.

Five kinds of scaffolds support the attachment of MC3T3 cells with spreading morphology (Figures 7(a)–7(e)). MTT analysis shows that cell proliferation increased with increased time of culture. There is no obvious difference among the five scaffolds in the first three days. However, after cultured for 7 days, the proliferation on the PLGA/CS and NMBG-PLGA/CS scaffolds was significantly higher than on the pure CS scaffolds (Figure 8(a)). The ALP activity of cells on the PLGA/CS and NMBG-PLGA/CS scaffolds was obviously higher than that on pure CS scaffolds, but there were no obvious differences among the three groups of NMBG-PLGA/CS scaffolds (Figure 8(b)).

### 3.5. Loading and *In Vitro* Release of IBU for NMBG-PLGA/CS Scaffolds

**Figure 9:**
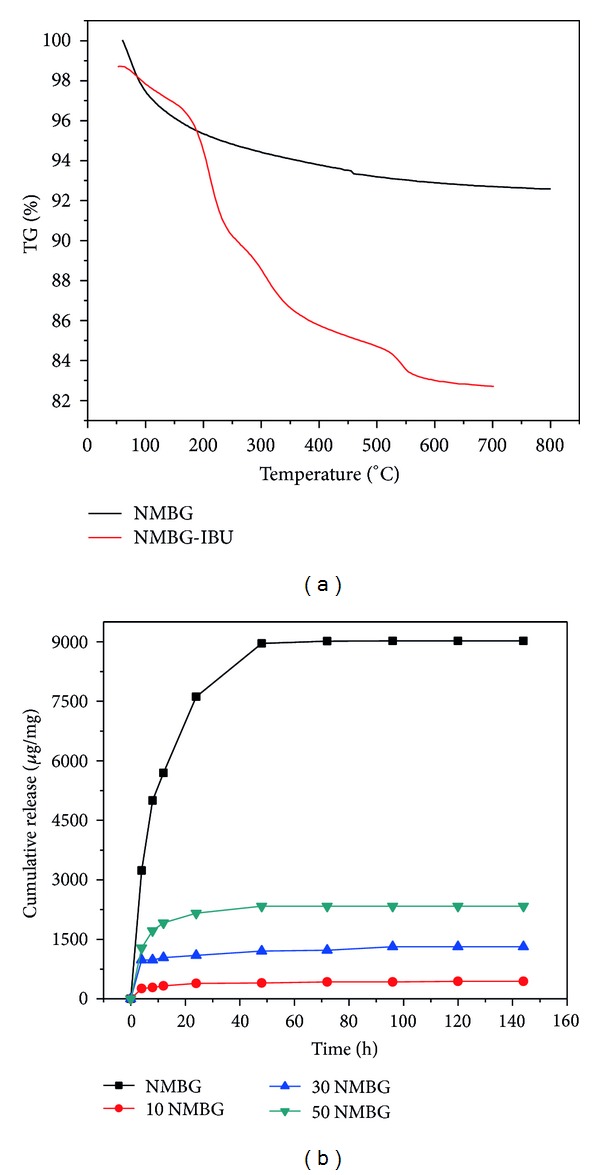
(a) TG analysis of IBU loading and (b) cumulative release of IBU from NMBG-PLGA/CS scaffolds.

The TG analysis suggests that the loading efficiency and amount of IBU in the NMBG powders are about 9.8% and 98 mg IBU/g NMBG (Figure 9(a)). The release amount of IBU increases with the increase of NMBG contents. Within the first 8 h, three NMBG-PLGA/CS scaffolds had a burst release and after 24 h, all scaffolds maintained a sustained release of IBU (Figure 9(b)).

## 4. Discussion

In this study, multifunctional NMBG-PLGA/CS scaffolds were successfully prepared for bone tissue engineering, which had been proved to possess improved mechanical strength, apatite-mineralization activity, and cytocompatibility as well as drug-delivery property, compared to pure CS and MBG scaffolds. The study indicates that the current method by introducing NMBG particles into the PLGA to coat CS scaffolds is a useful approach to construct multifunctional scaffolds to overcome the shortcomings of most bioceramic scaffolds in mechanical strength, bioactivity, and drug-delivery property. The method can be also applied to coat other kinds of scaffolds, such as *β*-tricalcium phosphate and hydroxyapatite.

It was found that the addition of NMBG in the PLGA/CS scaffolds had no obvious effect on the morphology of the macropore network, and the macropore size remained at a range between 300 and 500 *μ*m which can benefit cell ingrowth and nutrient transportation [27]. In addition, the compressive strength of NMBG-PLGA/CS scaffolds was significantly improved compared to pure CS scaffolds and similar to PLGA/CS scaffolds, indicating that PLGA modification plays a major role to enhance the mechanical strength of CS scaffolds. The compressive strength of NMBG-PLGA/CS scaffolds was around 2–2.5 MPa, which was higher than that of the PDLLA-modified CS scaffolds (1.45 MPa) [22], and was within the range of human cancellous bones (2–12 MPa) [25].

Previous studies suggest that apatite mineralization of biomaterials plays an important role to maintain the bioactive interface between materials and host bone tissues and the formed bone-like apatite could stimulate the proliferation and differentiation of osteoblasts [28–30]. In this study, after incorporation of NMBG particles in the scaffolds, the apatite-mineralization ability of the scaffolds was improved remarkably, indicating the stimulatory effect of NMBG particles on the apatite mineralization in SBF. To our knowledge, the typical mechanism of apatite mineralization on biomaterial surfaces mainly involves two important points [7, 31, 32]. Firstly, the release of Na^+^ and/or Ca^2+^ from the biomaterials that induces the formation of the negative surface with OH^−^ groups, providing the nucleation sites for apatite mineralization [33]. In this case, the NMBG had high surface area, which released Ca^2+^ quickly and induced large numbers of OH^−^ groups to deposit on the surface of scaffolds, further promoting the formation of apatite in SBF. Secondly, the surface microstructure had an impact on the bioactivity of biomaterials [34], while in our experiment, the NMBG particles attached on the pore walls increased the surface roughness of the scaffolds, which might offer more nucleation sites for apatite formation than PLGA/CS and pure CS scaffolds. It was demonstrated in Figure 6 that the P concentration decreased continuously as the formation of apatite consumed large amounts of P ions, while the Ca and Si ions concentrations kept increasing because of degradation of scaffolds, which released Si and Ca. Since part of the Ca ions was involved in apatite deposition process, the increase kinetics of Ca ions was slower than Si ions. PLGA/CS scaffolds showed the slowest release of Ca and Si, indicating that the polymer had served as a shield on the scaffold surface, and impeded the apatite formation. However, after NMBG was added in the scaffolds, the shield effect was decreased and Ca and Si ions were easily released.

Further cell experiments were carried out to investigate the effect of NMBG particles on the cell response in the scaffolds. The results indicate that the NMBG-PLGA/CS scaffolds have improved cell proliferation and earlier differentiation of MC3T3 than pure CS scaffolds, but similar to PLGA/CS scaffolds. It is known that the surface microstructure and the released ions from biomaterials are two major factors that influence the cell response [32, 35, 36]. Previous studies have shown that, at a certain concentration range, Ca and Si ions can stimulate cell proliferation and differentiation [32, 37]. In this study, although the incorporation of NMBG leads to increased Ca and Si ionic concentrations, it seems that the effect of increased Ca and Si concentrations have no significant effect on cell proliferation and ALP activity of MC3T3 cells. However, the PLGA modification seems to play the key role to enhance the cytocompatibility of scaffolds. We speculated that PLGA coatings on pure CS scaffolds created a more stable surface for cell adhesion, proliferation, and early differentiation [6, 11, 33].

Bacterial infection has been a serious problem in bone related surgery, causing complication and even osteonecrosis in the lesions. Traditional treatments such as wound drainage and implant removal result in much pain of additional surgical intervention to patients [15]. To deal with this problem, an efficient, controllable, and nontoxic local drug release system is needed. In our study, the NMBG particles with mesoporous channel structures can serve as drug carriers. Since pure CS scaffolds lack nanopore structure, they cannot be used as drug-delivery system. However, in this study, the mesopores of NMBG particles had the distinct ability for loading IBU. In addition, with the relatively slow degradation rate of PLGA coating on the surface, drugs can be released gradually and continuously. It turned out that the NMBG-PLGA/CS scaffolds have sustained drug release property. Therefore, the NMBG-PLGA/CS scaffolds combine the bioactivity and drug-delivery property and might be used for bone tissue engineering.

## 5. Conclusion

Multifunctional NMBG-PLGA/CS scaffolds were successfully prepared by coating sintered pure CS scaffolds with NMBG incorporated PLGA solution. The NMBG-PLGA/CS scaffolds possess macroporous structures (300–500 *μ*m) and their pore walls contain nanosized mesoporous bioactive glasses (mesopore size: 3.5 nm). Compared with CS scaffolds and pure PLGA-coated CS scaffolds, the mechanical strength and mineralization ability of NMBG-PLGA/CS scaffolds were greatly improved due to the addition of NMBG particles. The prepared NMBG-PLGA/CS scaffolds enhanced the proliferation and early cell differentiation of MC3T3 cells as compared to CS scaffolds. Moreover, the mesopore channels inside the NMBG particles offer NMBG-PLGA/CS scaffolds sustained drug-delivery ability for dealing with potential bacterial infection. All these results indicate that the NMBG-PLGA/CS scaffolds may be used as a multifunctional platform for bone tissue engineering.
